# Efficient genome editing using CRISPR/Cas9 ribonucleoprotein approach in cultured Medaka fish cells

**DOI:** 10.1242/bio.035170

**Published:** 2018-08-02

**Authors:** Qizhi Liu, Yongming Yuan, Feng Zhu, Yunhan Hong, Ruowen Ge

**Affiliations:** Department of Biological Sciences, National University of Singapore, 117543, Singapore

**Keywords:** Genome editing, CRISPR/Cas9, Medaka, RNP, Electroporation

## Abstract

Gene editing with CRISPR/Cas9 is a powerful tool to study the function of target genes. Although this technology has demonstrated wide efficiency in many species, including fertilized zebrafish and medaka fish embryos when microinjected, its application to achieve efficient gene editing in cultured fish cells have met some difficulty. Here, we report an efficient and reliable approach to edit genes in cultured medaka (*Oryzias latipes*) fish cells using pre-formed gRNA-Cas9 ribonucleoprotein (RNP) complex. Both medaka fish haploid and diploid cells were transfected with the RNP complex by electroporation. Efficient gene editing was demonstrated by polymerase chain reaction (PCR) amplification of the target gene from genomic DNA and heteroduplex mobility assay carried out with polyacrylamide gel electrophoresis (PAGE). The heteroduplex bands caused by RNP cleavage and non-homologous end joining could be readily detected by PAGE. DNA sequencing confirmed that these heteroduplex bands contains the mutated target gene sequence. The average gene editing efficiency in haploid cells reached 50%, enabling us to generate a clonal cell line with *ntrk3b* gene mutation for further study. This RNP transfection method also works efficiently in diploid medaka cells, with the highest mutation efficiency of 61.5%. The specificity of this synthetic RNP CRISPR/Cas9 approach was verified by candidate off-target gene sequencing. Our result indicated that transfection of pre-formed gRNA-Cas9 RNP into fish cells is efficient and reliable to edit target genes in cultured medaka fish cells. This method will be very useful for gene function studies using cultured fish cells.

## Abstract (precis)

**Summary:** In this paper, we report an efficient genome editing method for cultured medaka fish cells using pre-formed CRISPR/Cas9 RNP. This method will be very useful for gene function studies using cultured fish cells.

## INTRODUCTION

The CRISPR/Cas9 gene editing technology was originally derived from the bacterial adaptive immune system, using a guide RNA activated Cas9 nuclease to cleave double-stranded DNA targets (Horvath and Barrangou, 2010). The CRISPR/Cas9 approach has been widely used as a simple and precise genome editing tool for genetic study (Brouns et al., 2008; Kleinstiver et al., 2015; Ran et al., 2013; Wiedenheft et al., 2012). The target cleavage site depends on the 20-nt sequence on the gRNA followed by NGG PAM (protospacer adjacent motif). After formation of a double strand break (DSB), the host will repair the genome by non-homologous end joining (NEHJ) or homology-directed repair (HDR). Upon NEHJ, endogenous DNA repair machinery attempts to fix the DSB, but it often leads to random insertion and deletion (indel), resulting in frameshift and gene knockout (Brouns et al., 2008). On the other hand, for HDR, a DNA cassette flanked by homologous arms is inserted into the DSB, causing knockin (Ran et al., 2013).

The CRISPR-Cas9 genome editing system has been widely used in many species, including various teleost fish species such as the model fish zebrafish (Chang et al., 2013; Hwang et al., 2013; Jao et al., 2013) and medaka fish (Ansai and Kinoshita, 2014). This gene editing system was also successfully used in food fish species such as tilapia (Feng et al., 2015; Li et al., 2014) and Atlantic salmon (Edvardsen et al., 2014). By microinjecting the synthesized single guide RNA (sgRNA) and mRNA encoding Cas9 nuclease into the fish embryos, the CRISPR/Cas9 system was reported to be a powerful tool in teleost fish genome editing.

Despite the successful application of CRISPR/Cas9 gene editing in fish embryos via microinjection, its application in cultured fish cells has been limited, possibly due to the low efficiency of introducing CRISPR/Cas9 elements into fish cells. Moreover, the fish spawning season is very short for most aquatic fish species compared to model fish, thus the availability of fish embryo is limited. Hence, to fully understand the function of fish genes, a cost-efficient loss-of-function gene editing method in fish cells is needed. To date, only two recent reports have presented successful gene knockout in cultured fish cells using the CRISPR/Cas9 method. Dehler et al. reported successful editing of a stably integrated EGFP gene in cultured Chinook salmon CHSE cells through stable overexpression of a nuclear localized Cas9 (nCas9) and subsequent transient transfection of sgRNA targeting EGFP (Dehler et al., 2016). The EGFP gene was disrupted in 34.6% of cells in this case. In another example, the JAM-A gene was knocked out using CRISPR/Cas9 method in cultured grass carp kidney cells via transfection of an all-in-one plasmid vector expressing both Cas9 and gRNA simultaneously (Ma et al., 2018). The gene editing efficiency in this work was not reported.

During our work to study gene function during viral infection using cultured fish cells, we encountered difficulties in generating gene knockout mutant cell lines using either plasmid or lentivirus mediated gRNA and Cas9 expression systems (data not shown). Possible reasons were speculated for the difficulty encountered in applying the CRISPR/Cas9 gene editing technique in cultured fish cells. First, there is a lack of well characterized and efficient polymerase III promoter (such as medaka U6 promoter) that can work well in medaka fish cells. Mammalian U6 promoter does not work well in medaka fish cells. Second, transiently transfecting synthetic gRNA into medaka fish cells is also ineffective, likely due to the difficulty to transfect fish cells and fast degradation of gRNA in culture media. Here, we report a simple and efficient CRISPR/Cas9 gene editing method for cultured medaka fish cells by electroporation of pre-formed gRNA/Cas9 ribonucleoprotein (RNP) complex. This method eliminates the need to (1) identify an effective polymerase III promoter that works well in the particular fish species for sgRNA expression and (2) construct sgRNA expressing recombinant plasmid or lentivirus.

## RESULTS AND DISCUSSION

### RNP-mediated CRISPR/Cas9 gene editing in cultured medaka cells using a reporter plasmid pCut

**Fig. 1.**
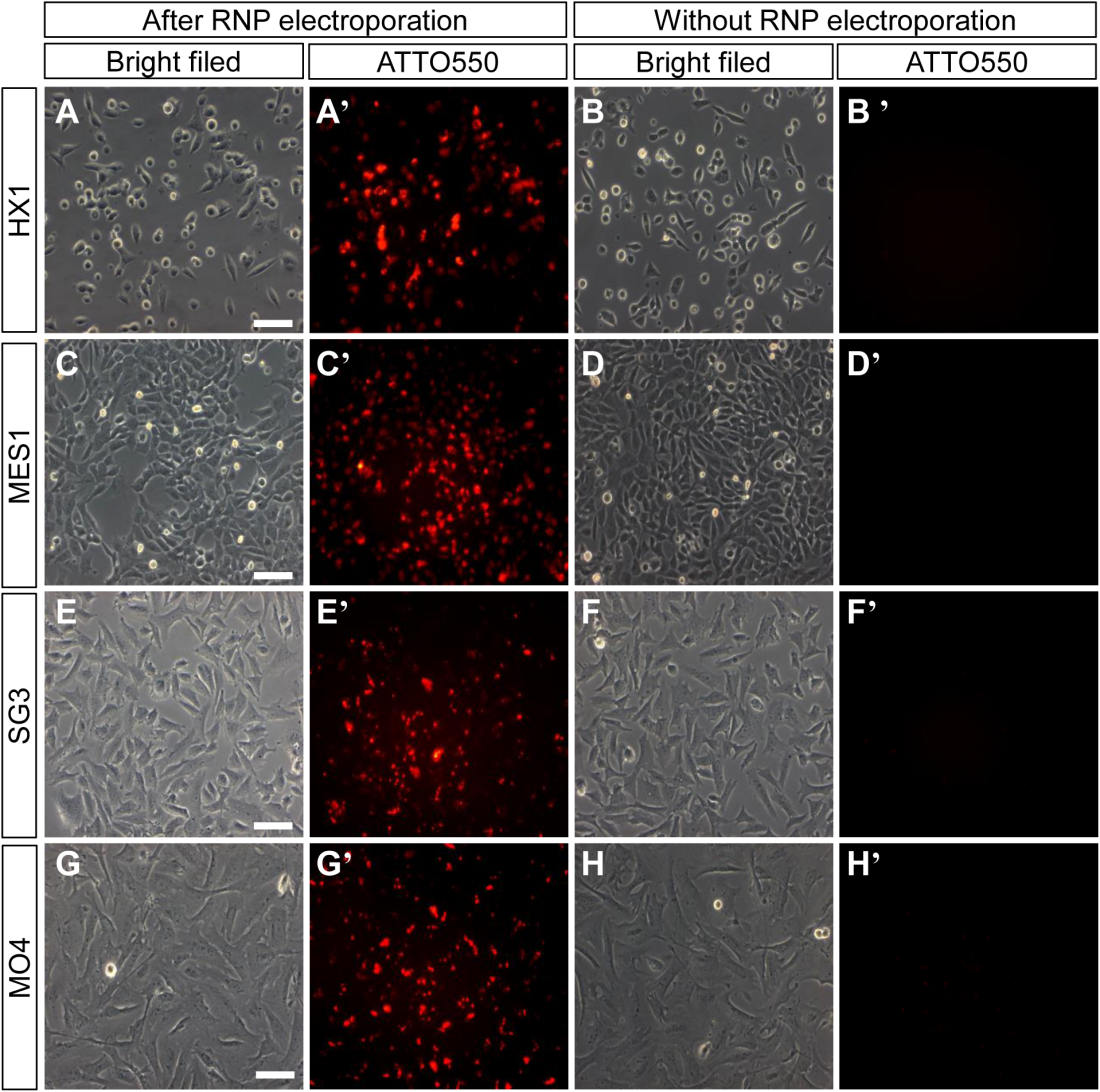
**Microscope photographs of medaka cells transfected with RNP by electroporation.** The RNP containing ATTO550-conjugated tracrRNA was transfected into medaka cells by electroporation (A,A′,C,C′,E,E′,G,G′). After 24 h, the cells were monitored under fluorescent microscopy to check the transfection efficiency. Comparing to the cells without electroporation (B,B′,D,D′,F,F′,H,H′), the red fluorescent signal was detected in HX1 (A′), MES1 (C′), SG3 (E′) and MO4 (G′) cells transfected with RNP-ATTO550 by electroporation. Scale bars: 20 μm.

After failed attempts to achieve gene editing using expression plasmids or sgRNA, we tried the electroporation of the Cas9:tracrRNA:crRNA RNP complex method in cultured medaka fish cells. Two annotated medaka genes, *sytl5* and *tmem104,* were selected as gene editing targets using this RNP approach. The crRNA targeting either exon 1 or exon 2 of these genes were designed according to the CCTop - CRISPR/Cas9 target online predictor (Stemmer et al., 2015). The crRNAs were annealed with ATTO 550 labelled universal tracrRNAs and subsequently incubated with recombinant Cas9-3NLSnuclease to form the RNP complex. RNP complexes were electrophoresed into cultured medaka cells together with carrier DNA. We found that electroporation can achieve the highest transfection efficiency for four medaka cell lines, including haploid and diploid embryonic stem cells lines HX1 and MES1, spermatgonia stem cell line SG3 and ovary cell line MO4 (Fig. 1). At 1 day post transfection (dpt), more than 90% of cells had the red fluorescence of ATTO 550.

**Fig. 2.**
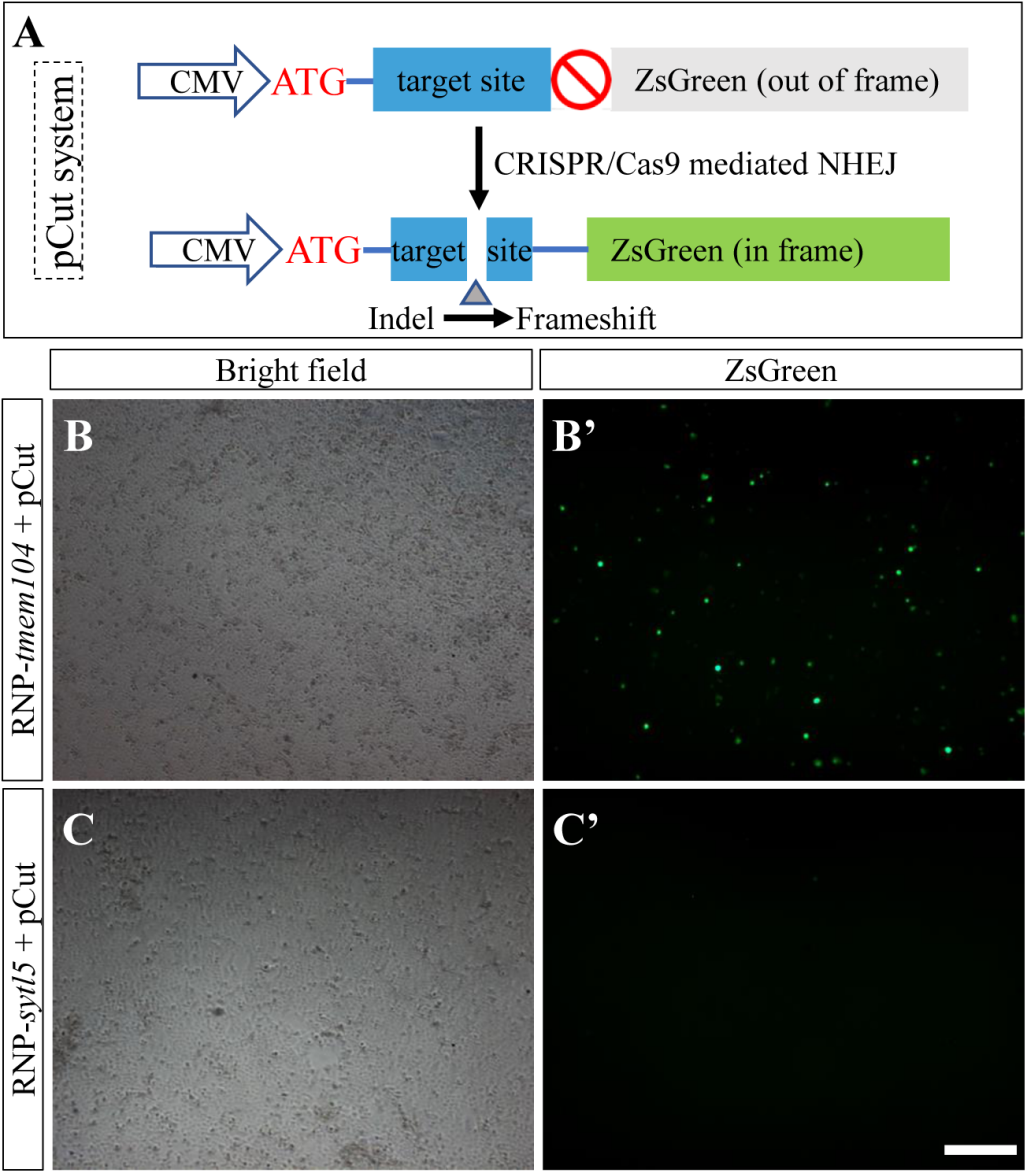
**Monitoring the efficacy and specificity of CRISPR/Cas9 RNP-mediated cleavage in HX1 cells with pCut system.** pCut vector containing the target sequence of *tmem104* was transfected together with RNP-*sytl5* and RNP-*tmem104* into HX1 cells with electroporation. After 3 days of culture, the green fluorescent signal was detected, indicating the RNP cleaved the target sequence. (A) Schematic representation of the pCut system. ZsGreen is out of frame due to the insertion of 23 bp target sequence after start codon. Once the CRISPR/Cas9 RNP successfully generated indels within the target site, the reading frame shift led to a correct expression of ZsGreen, which was detected under fluorescent microscopy. (B-C′) Bright field and fluorescent microscopy photographs of HX1 cells transfected with pCut plus RNP-*tmem104* (B,B′) and RNP-*sytl5* as control (C,C′) respectively. Green signal was only detected in (B′), indicating that the pCut vector was specifically cleaved by RNP-*tmem104* containing the identical target sequence. Scale bar: 300 µm.

To monitor the RNP mediated gene editing efficacy and specificity, we constructed the pCut reporter plasmid that contains the identical crRNA-*tmem104* target sequence. In pCut, a *tmem104* target sequence was inserted between Cytomegalovirus (CMV) promoter and ZsGreen, causing a frame shift of the fusion protein; hence no green fluorescence (Fig. 2A). After co-transfecting pCut and RNP-tmem104 into cells, if the RNP cleave the *tmem104* target sequence in pCut and generate various indels, some of the indels would result in corrections of the reading frame, leading to translation of ZsGreen protein and emission of green fluorescence (Fig. 2B′). In comparison, there is no green fluoresce signal in control cells which were transfected with pCut and RNP-sytl5, since the RNP-sytl5 cannot cleave pCut (Fig. 2C′). These results demonstrate that the RNP-tmem104 is effective and specific in cleaving its target sequence.

### Endogenous gene editing in cultured medaka haploid ES cells

After validating the efficacy and specificity of RNP, we selected two endogenous genes to target using medaka haploid ES cells HX1. To validate the RNP-mediated gene editing, genomic DNAs were extracted from each RNP-electroporated cell pool at 7 dpt, followed by polymerase chain reaction (PCR) amplification and DNA heteroduplex mobility assay (HMA) with polyacrylamide gel electrophoresis (PAGE). HMA has been shown to be an efficient method to detect and screen small gene sequence alterations in the genome, allowing for direct cloning and sequencing of the DNA mutations (Chen et al., 2012).

**Fig. 3.**
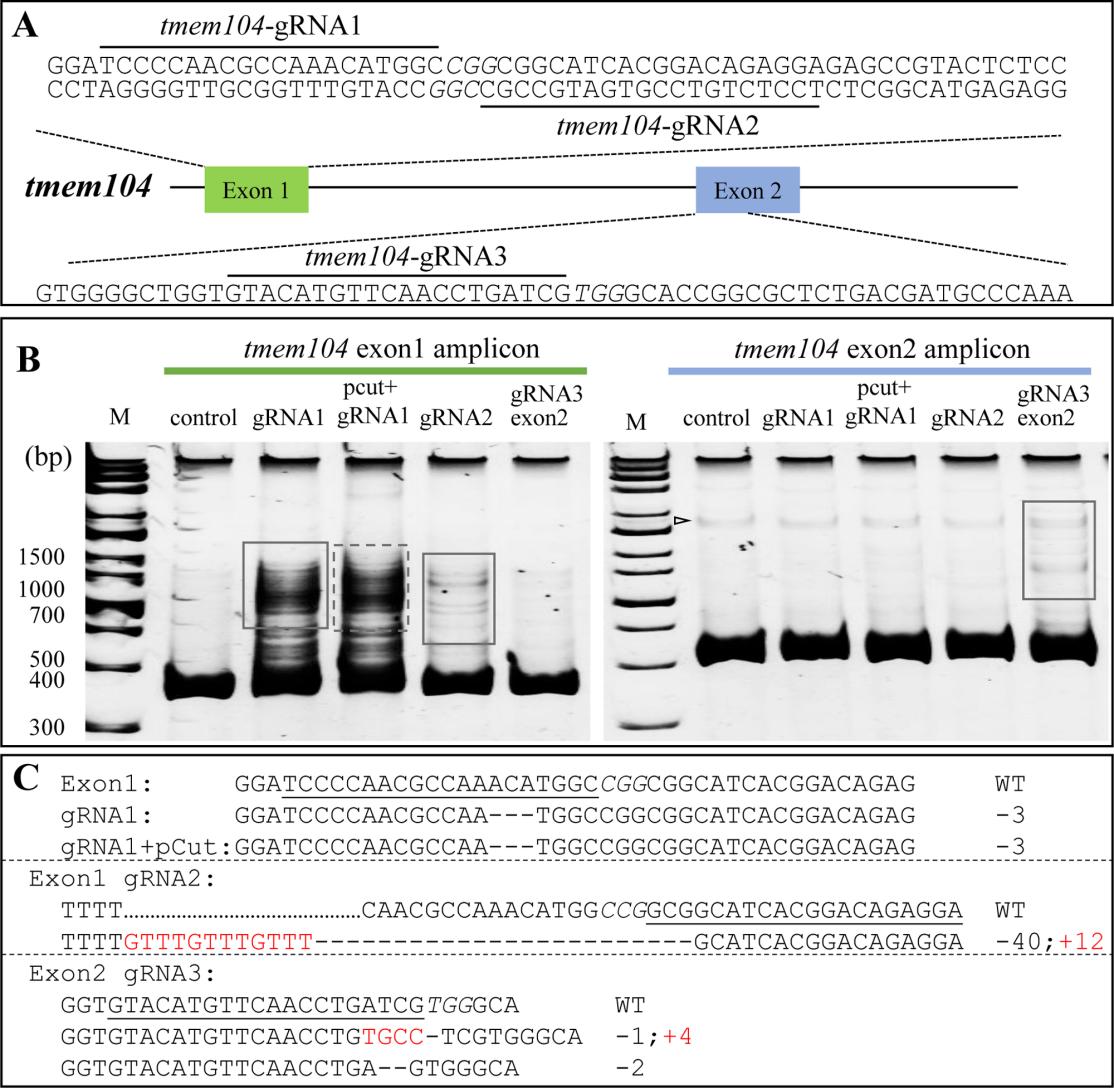
**RNP efficiently generated** **indels at *tmem104* in haploid medaka fish cells.** RNP targeting *tmem104* exon 1 and exon 2 was transfected into HX1 cells. After incubating for 7 days, the genomic DNA was extracted for PCR and amplicons were separated with PAGE, giving a main band of homoduplex and upper bands consisting of heteroduplex or homoduplex. The upper bands were recovered for sequencing to validate the mutation. To test whether the pCut affects the cleavage efficient of RNP, HX1 was transfected with pCut plus RNP as control. (A) RNP target sites in *tmem104*. Targets are highlighted with lines and PAMs are Italic. *Tmem104*-gRNA1 and 2 target the site in exon 1. *Tmem104*-gRNA3 targets sequence in exon 2. (B) Heteroduplex assay of cells transfected with RNPs targeting *tmem104* exon 1 (left) and exon 2 (right) by PAGE gel. Control lanes are amplicons from DNA of wild-type genome, exon 1 target fragment on the left and exon 2 target fragment on the right. Triangle indicates the background heteroduplex band present in all samples including the control. Boxed areas indicate the heteroduplex DNA bands that are results from the indels from gRNP. (C) Sequences of *tmem104* mutations after introducing RNP containing related gRNA.

In cells transfected by RNP-tmem104, although wild-type cells presented some background heteroduplex bands, likely due to SNPs within the target region (Fig. 3B, triangle), heteroduplex or homoduplex DNA bands caused by indels can be clearly distinguished (Fig. 3B, left, boxed). The pCut plasmid did not affect the cleaving efficiency of RNP-tmem104 (Fig. 3B, lane gRNA1 vs pCut+gRNA1, dash line box). Furthermore, amplification of the non-target fragment did not show any heteroduplex (Fig. 3B). For example, when the cells were transfected with RNP-tmem104 exon 1 gRNAs, the DNA fragment in exon 2 was amplified as control (Fig. 3B, left). No heteroduplex was formed in the amplified exon 2 fragment as expected. Meanwhile, heteroduplex bands were detected in the DNA fragment amplified from exon 2 in cells transfected by RNP targeting exon 2 (Fig. 3B, right, boxed). These results demonstrate the specificity of RNPs. The heteroduplex bands were recovered for PCR amplification and subcloned into plasmid for DNS sequencing. For each gRNA, 10 plasmid clones were sequenced to identify the mutation. Examples of indels caused by RNP are shown in Fig. 3C. It was noted that different gRNA has different efficacy in generating indels as expected. For tmem104 exon 1, gRNA2 and gRNA3 is not as effective as gRNA in generating indels. As for exon 2 region, gRNA3 is more effective than gRNAs 1 or 2.

**Fig. 4.**
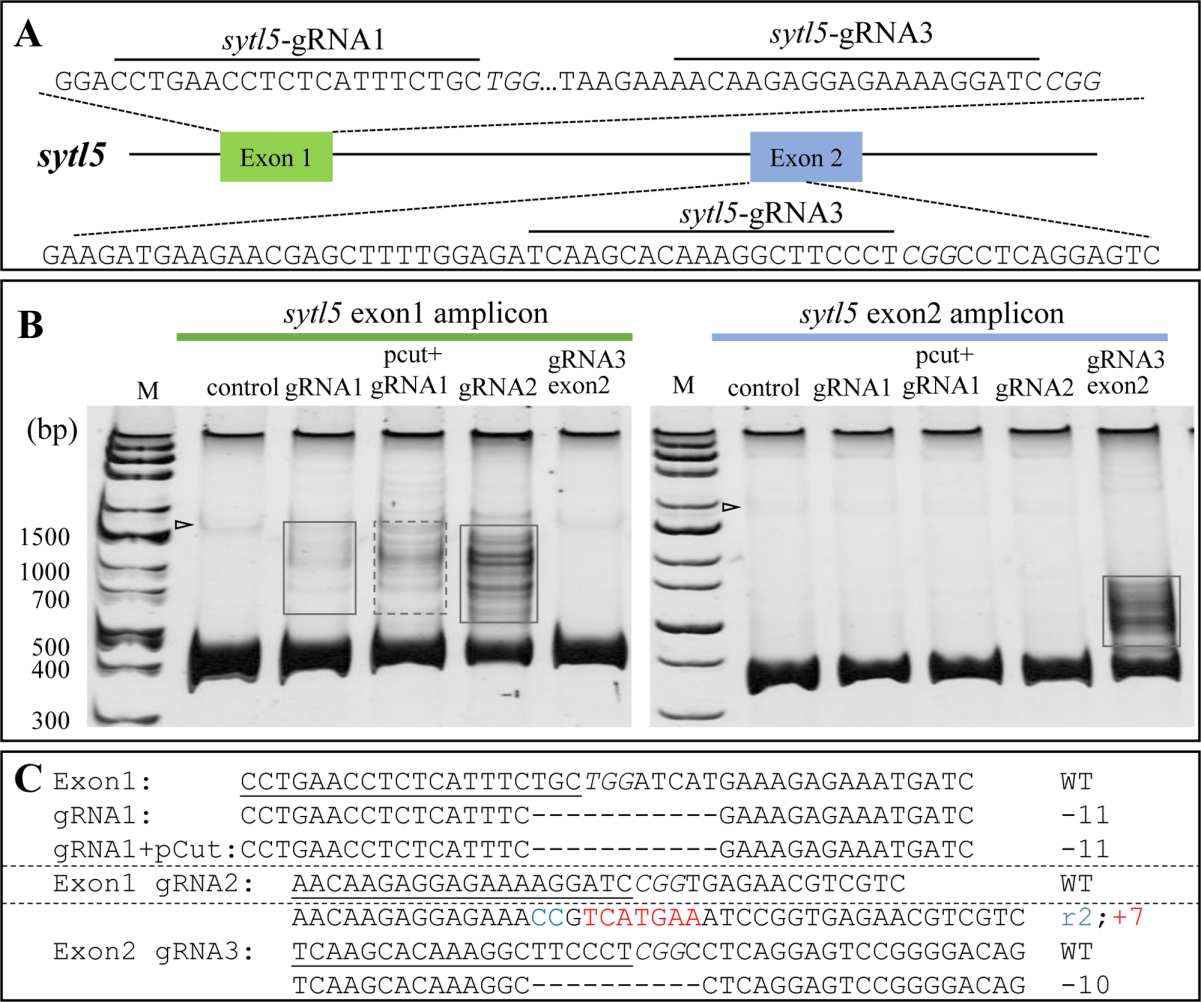
**RNP efficiently generated indels at *sytl5* in haploid medaka fish cells.** RNP targeting *sytl5* exon 1 and exon 2 was transfected into HX1 cells respectively. After incubating for 7 days, the genomic DNA was extracted for PCR and amplicons were separated by PAGE, presenting a main band of homoduplex and upper bands consisting of heteroduplex. The upper bands were recovered for sequencing to validate the mutation. To test whether the pCut affects the cleavage efficient of RNP, HX1 was transfected by pCut together with RNP as control. (A) RNP target sites in *sytl5*. Targets are highlighted with lines and PAMs are in Italic. (B) Heteroduplex assay of cells transfected with RNPs targeting *sytl5* exon 1 (left) and exon 2 (right) by PAGE gel. The heteroduplex bands in amplicon from wild type cells are indicated with a triangle. Heteroduplex bands in amplicons from RNP transfected cells are boxed. (C) Sequences of *sytl5* mutations from the recovered bands (boxed in B) after introducing RNP containing related gRNA.

Similarly, heteroduplex bands and indels were readily detected from RNP-sytl5 transfected cell pools (Fig. 4). Consistently, heteroduplex bands were only detected in the PCR amplicons from RNPs targeting the correct target sequence (Fig. 4B, left, boxed), but not from wild-type genome as shown in the control lane. Using the online tool TIDE (Tracking of Indels by Decomposition), we analyzed the directly sequenced heteroduplex DNA mixture. An indel rate up to 50% was observed. Taken together, these results indicate that the RNP transfection method can efficiently and specifically accomplish gene editing in medaka haploid ES cells.

### RNP transfection can readily generate mutant cell clones

**Fig. 5.**
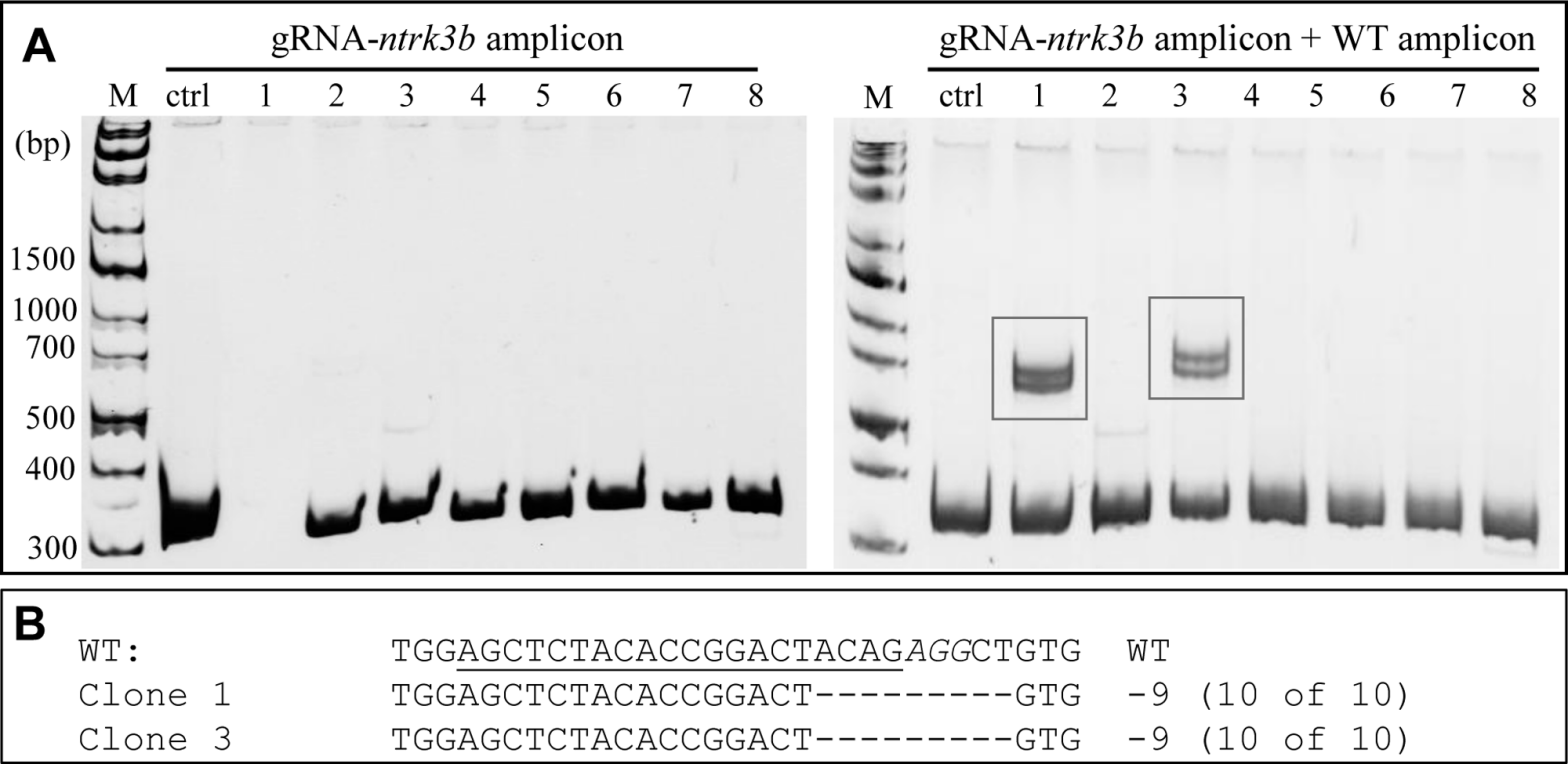
**Generation of individual cell clones with target gene edited.** RNP targeting *ntrk3b* was introduced into HX1 cells with electroporation. After 7 days of culture, the cells were diluted and cultured for single colony picking. The DNA from each cell clone was extracted for PCR amplification and the amplicons were analyzed with PAGE. Meanwhile, the amplicons generated with the DNA template from each clone and the wild-type cells were mixed at a ratio of 1:1 and analyzed with PAGE after annealing. (A) Heteroduplex assay of single-cell clone. The homoduplex band of amplicon from a single clone was detected with PAGE electrophoresis (left). After annealing with the amplicon from DNA template of wild-type cells, the heteroduplex bands were detected (boxed), indicating the mutation in cell clone of 1 and 3 (right). (B) Sequences of *ntrk3b* mutation in single-cell clone 2 and 4. The PCR products amplified from the DNA template of each cell clone was ligated into cloning vector and transformed into *E.coli* for colony picking. In total, six colonies from each transformed strain were picked for plasmid extraction and sequencing. Results showed all of the colonies contained the mutated DNA fragment compared to the wild type, indicating the success of editing the target gene.

Although HX1 cells are haploid cells, the cultured cells frequently contain a mixture of haploid and diploid cells due to spontaneous duplication of chromosomes during cell proliferation. Using the RNP transfection method, we targeted a third gene, *ntrk3b*, in HX1 cells. Through dilution and subculture, single-cell clones were picked from the transfected cell pool. Mutation screening of individual cell clones using genomic PCR and HMA was carried out. From the first round of HMA, no heteroduplex band was detected (Fig. 5A, left), indicating the possible existence of only homoduplex of wild-type gene or mutated gene. To identify whether the homoduplex contains the mutated sequence, the PCR amplicon from wile-type cells was mixed with PCR amplicon from RNP mutated cell clones and annealed for PAGE analysis. As shown in Fig. 5A (right), cell clone 1 and cell clone 3 contained mutated *ntrk3b* gene, since heteroduplex bands was detected. DNA sequencing confirmed that a 9-base pair deletion was detected in both clones (Fig. 5B). These results indicate that mutated haploid cell clones can be readily generated by RNP transfection.

### Endogenous gene editing in cultured medaka diploid cell lines

**Fig. 6.**
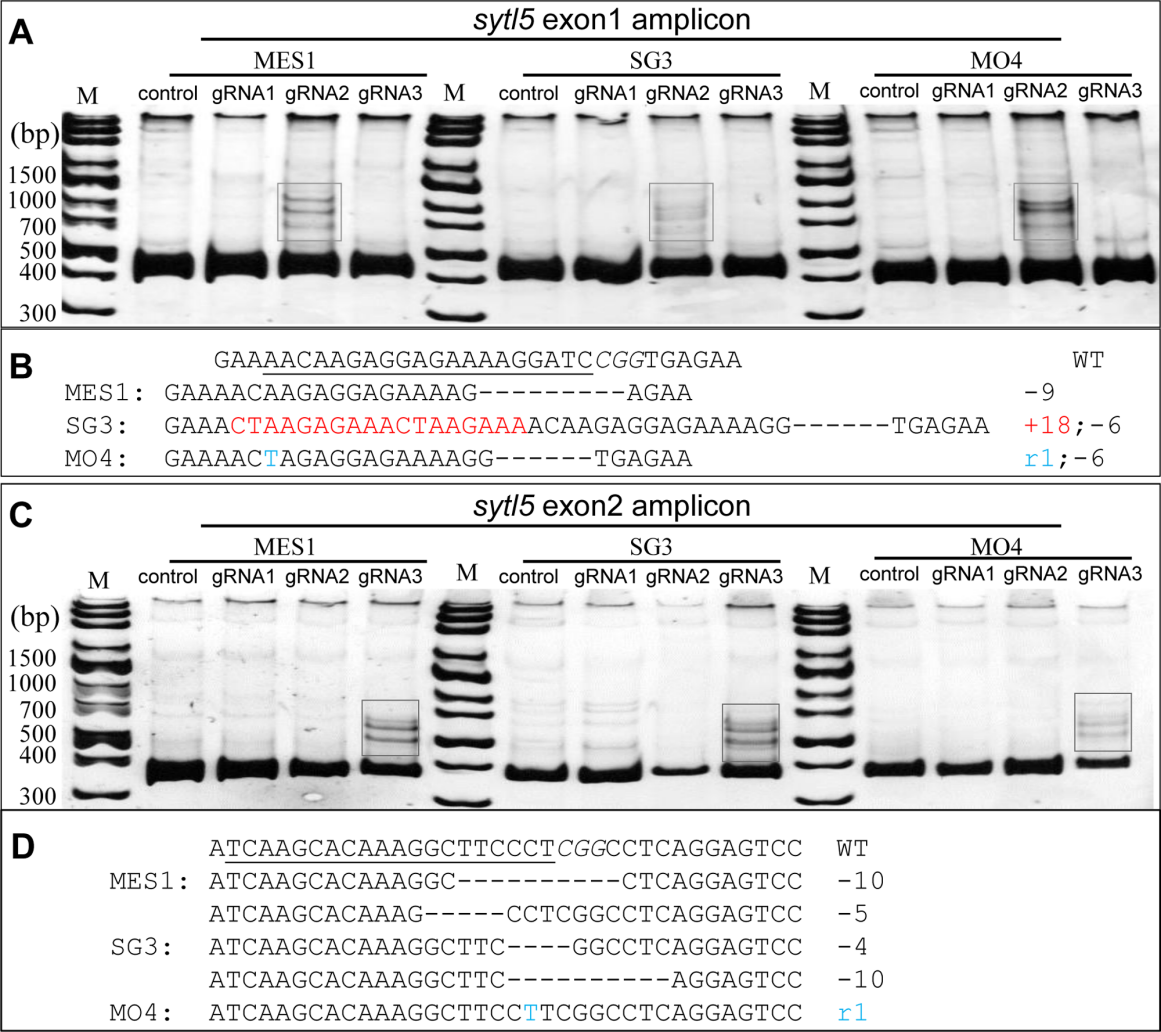
**The RNP transfection method can efficiently generate indels at *styl5* exon** **1 and exon** **2 in medaka fish cell lines MES1, SG3 and MO4.** (A) Heteroduplex assay of the amplicon of *sytl5* exon 1 in MES1, SG3 and MO4 transfected with RNP. (B) Sequences of *sytl5* mutation related to *sytl5* gRNA2 (boxed in A). (C) Heteroduplex assay of the amplicon of *sytl5* exon2 in MES1, SG3 and MO4 transfected with RNP. (D) Sequences of *sytl5* mutation related to *sytl5* gRNA3 respectively (boxed in C).

**Fig. 7.**
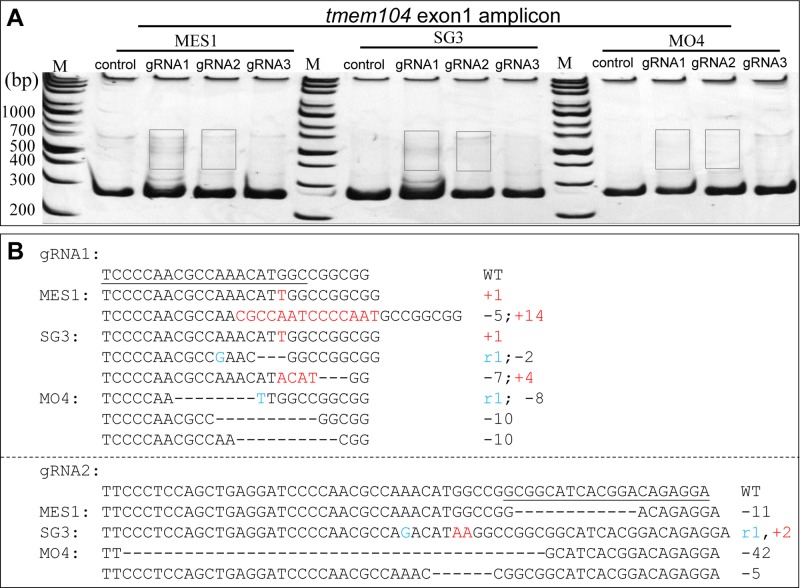
**The RNP transfection method can efficiently generate indels at *tmem104* exon** **1 in medaka fish cell lines** **MES1, SG3 and MO4.** (A) Heteroduplex assay of the amplicon of *tmem104* exon 1 in MES1, SG3 and MO4 transfected with RNP. (B) Sequences of *tmem104* mutations related to gRNA1 and gRNA2 respectively (boxed in A).

To determine whether the RNP method can induce indels in medaka diploid cell lines, RNP targeting *sytl5* and *tmem104* were transfected into MES1, SG3 and MO4 cells with electroporation. Consistent with the HX1 cells, heteroduplex bands generated by gRNAs were detected in PAGE gel (Figs 6 and 7). Subsequent sequencing of the recovered bands confirmed the indel in cells (Figs 6B,D and 7B). The highest mutation efficiency reached 61.5% in MO4 cells calculated by TIDE.

### Specificity of RNP method in medaka cells

The specificity of the RNP method is supported by the above results that RNP did not cleave the non-target sequence in pCut and endogenous genes (Figs 2, 3 and 4). To further investigate the off-target rate, we examined three candidate off-target sequences for the three genes mutated above: *sytl5*, *ntrk3b* and *tmem104*, by using the CCTop - CRISPR/Cas9 target online predictor. Sequences with three or four base mis-match from each gRNA were selected from the medaka genome sequence (Table S1). DNA fragments containing the candidate off-target sequences were amplified by PCR. After PAGE, the heteroduplex DNA bands were recovered for sequencing and the off-target events were counted using TIDE. The PAGE profile showed that there are some SNPs in the candidate sequences, but no significant difference of the heteroduplex band pattern between control and the RNP treated cells were observed (Figs S1 and S2). Subsequent sequencing confirmed that the enriched off-target events ranged from 0% to 8.1% (Figs S1 and S2).

It should be noted that the off-target efficiency detected in our study is actually an enriched efficiency, since the heteroduplex bands are separated from the main band by PAGE and enriched by PCR amplification. We tried direct sequencing of the PCR product from off-target region before PCR enrichment and the mutation could not be found (data not shown). So the actual off-target rate should be much lower than what we have presented in Figs S1 and S2.

In conclusion, we present here an efficient and reliable RNP transfection method to edit genes in cultured medaka fish cells using the CRISPR/Cas9 technology. Mutations in three medaka genes were efficiently obtained in both haploid and diploid medaka cell lines. In addition, pure *ntrk3b* mutant cell clones could be readily generated from medaka haploid HX1 cells. It is envisioned that homozygous mutant cell clones can also be readily generated from diploid fish cells. This method should be very useful for fish researchers to study gene function in cultured fish cells, overcoming the technical bottleneck of the lack of effective polymerase III promoter from different fish species and the low transfection efficiency of cultured fish cells. It also eliminated the need to obtain effective expression plasmid or virus to produce gRNA and Cas9 proteins in fish cells.

## MATERIALS AND METHODS

### Plasmid

The CRISPR/Cas9 mutation reporter plasmid pCut (Fig. 1A) was constructed with the backbone of pcDNA3.1. An ‘ATG’ start codon and a crRNA target sequence including PAM motif for medaka gene *tmem104* (TCCCCAACGCCAAACATGGCCGG) were inserted downstream of the CMV promoter between restriction sites of NheI and NotI. DNA sequence encoding ZsGreen was then inserted into the restriction sites of NotI and ApaI. ZsGreen DNAwas amplified with primer pairs of ZsgreenNotF (AGCGGCCGCACGCCCAGTCCAAGCAC) and ZsgreenApaR (AGGGCCCTTAGGGCAAGGCGGAGCCG) from pZsGreen template (Clontech, Palo Alto, CA, USA). Plasmid DNA used for electroporation was prepared using the Plasmid Midi-prep kit (Qiagene).

### Cell culture

Medaka fish cell lines were maintained in ESM4 at 37°C under ambient air as described (Hong et al., 2004, 1996, 1998; Yi et al., 2009, 2010). Cell lines used include haploid ES cell line HX1 (Yi et al., 2009), diploid ES cell line MES1 (Hong et al., 1996, 1998), spermatogonial stem cell line SG3 from the adult testis (Hong et al., 2004) and medaka ovary cell line MO4 (a cell line developed in our lab).

### RNP electroporation

Recombinant *S. pyogenes* Cas9 Nuclease 3NLS, crRNA, tracrRNA ATTO 550 and none-targeting carrier DNA were obtained from Integrated DNA Technologies (Skokie, IL, USA). To form crRNA: tracrRNA complex, 5 μl 200-μM crRNA and 5 μl 200-μM tracrRNA were mixed and heated at 95°C for 5 min. The mixture was then incubated at room temperature for 30 min. To form RNP complex, 2.1 μl DPBS, 1.2 μl crRNA: tracrRNA complex and 1.7 μl Cas9 nuclease (61 μM) were mixed and incubated at room temperature for 10-20 min. For RNP electroporation, 1 million medaka fish cells at 90% confluence were trypsinized and washed in 1 ml DPBS twice by spin-down and resuspension. Washed cells were suspended in 94 μl DPBS and 5 μl RNP complex and 1 μl 100 μM carrier DNA were added by gentle pipetting. The mixture was transferred to sterile electroporation cuvette with 0.2 cm gap (Bio-Rad Laboratories) and electroporated with 220 V and 5 ms by the Gene Pulser Xcell Electroporation Systems (Bio-Rad). At 24 h post electroporation (hpe), culture medium was changed and the cells were cultured for another 7 days before genotyping.

### Genotyping

For single-cell clone screening, the cells were directly lysed in culture plates for target fragment amplification with PCR. Briefly, cells cultured in 48-well plates were lysed by 50 µl of lysis buffer (10 mM Tris-HCl, pH 8.0, 1 mM EDTA, 1% SDS, 100 mg/ml proteinase K) at 50°C for 2 h. After briefly vortexing, 0.1-1 µl lysate containing genomic DNA was used for PCR in a reaction of 50 µl. Meanwhile, an appropriate amount of Tween 20 was added depending on the amount of SDS introduced into the system by the sample. The correlations between Tween 20 and SDS are: 2% Tween 20 for 0.05% SDS; 5% Tween 20 for 0.2% SDS.

To monitor the RNP cleavage efficacy based on heteroduplex DNA band, genomic DNA was extracted using commercial kit (K0512; Thermo Fisher Scientific) and 50 ng of genomic DNA was used for PCR amplification for 35 cycles (95°C for 30 s, 55°C for 30 s and 72°C for 30 s) with Dream-taq DNA polymerase (EP0703, Thermo Fisher Scientific). After PCR amplification, the product was denatured at 94°C for 3 min and slowly cooled down to room temperature to form heteroduplex or homoduplex. Subsequently, 4 µl of PCR products were separated on 8% polyacrylamide gels in TBE buffer using a Mini-Protean electrophoresis unit (Bio-Rad). Gels were stained by submerging in TBE buffer containing Gel Red for 20 min and documented on a bioimaging system (Vilber Lourmat, Collegien, France). DNA heteroduplex band can be identified using the PAGE pattern as previously described (Chen et al., 2012).

After imaging, the target DNA bands were cut from the gel under UV light and smashed in 10-20 µl miliQ water or Tris-EDTA (TE). After incubation overnight at room temperature, 1 µl of supernatant containing DNA was used as template for 30 cycles of PCR at the same conditions. PCR product were purified by gel extraction kit and ligated into pJet1.2 (K1231, Thermo Fisher Scientific) for *E. coli* transformation. A single colony was picked for plasmid extraction and sequencing.

### Analysis of gene editing efficiency and off-target events by TIDE assay

For TIDE analysis, 50 ng of purified PCR products were mixed with 5 pmol primer in a final volume of 15 μl and samples were subjected to Sanger sequencing. Sequencing chromatograms were analyzed by TIDE (Brinkman et al., 2014), and indel frequencies were determined by the addition of significant insertions and deletions (*P*<0.05).

### Microscopy

Microscopy was done on a Zeiss Axiovert2 inverted microscope equipped with a Zeiss AxioCam MRc digital camera and AxioVision 4 software as described (Yuan and Hong, 2016; Yuan et al., 2013).

## Supplementary Material

Supplementary information
