# Supplementary material for: Efficient genome editing using CRISPR/Cas9 ribonucleoprotein approach in cultured Medaka fish cells
Source: Biol Open. 2018 Aug 2;7(8):bio035170. doi: 10.1242/bio.035170 (PMC6124564; doi:10.1242/bio.035170)
Supplement: Supplementary information [file biolopen-7-035170-s1.pdf]

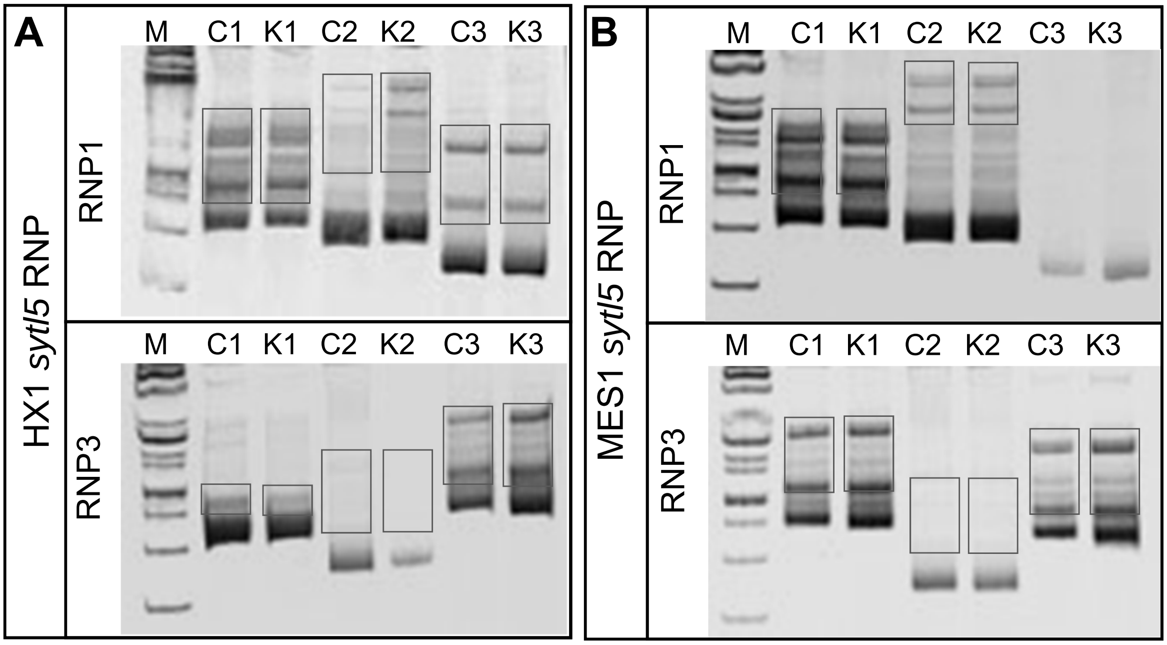

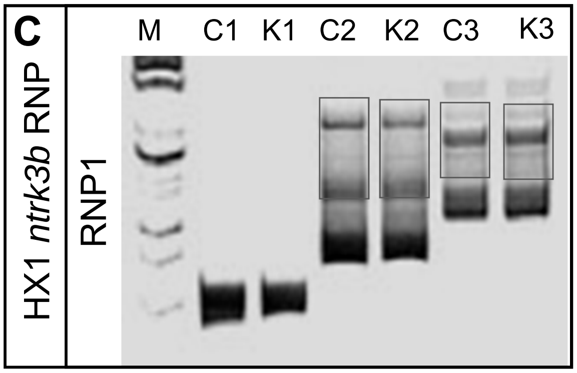

D

Sample	Off-target sequence	Enriched efficiency
ntrk3b-gRNA1-off1-HX1	AGACCTGCACTGGACTACAG	8.10%
ntrk3b-gRNA1-off2-HX1	GGCTCTGCTCTGGACTACAG	0.20%
ntrk3b-gRNA1-off3-HX1	TCCTCTACACATGACTACAG	5.90%
sytl5-gRNA1-off1-HX1	CCAGACTCCCTCATTTCTGC	0.00%
sytl5-gRNA1-off1-MES	CCAGACTCCCTCATTTCTGC	2.70%
sytl5-gRNA1-off2-HX1	CCCAAAACTGTCATTTCTGC	0.00%
sytl5-gRNA1-off2-MES	CCCAAAACTGTCATTTCTGC	2.70%
sytl5-gRNA1-off3-HX1	TCTTAACATTTCATTTCTGC	4.40%
sytl5-gRNA1-off3-MES	TCTTAACATTTCATTTCTGC	1.60%
sytl5-gRNA3-off1-HX1	CCGAGCACAAAGGCTACCCT	8.60%
sytl5-gRNA3-off1-MES	CCGAGCACAAAGGCTACCCT	0.00%
sytl5-gRNA3-off2-HX1	TCAGGCAGAAATGCCTCCCT	0.00%
sytl5-gRNA3-off2-MES	TCAGGCAGAAATGCCTCCCT	1.00%
sytl5-gRNA3-off3-HX1	CCAATCACAGAGGCTTTCCT	4.80%
sytl5-gRNA3-off3-MES	CCAATCACAGAGGCTTTCCT	5.40%

Fig. S1. Analysis of the enriched off-target events for *styl5* and *ntrk3b* with PAGE and TIDE. The DNA of wild type and RNP treated cells are extracted. The off-target candidate genome sequences are selected and amplified with PCR. The DNA fragment of three off-target candidate sequence was amplified respectively from the template of wild type control (C1-C3) and RNP knockout cells (K1-K3). After PAGE separation, enriched heteroduplex bands (boxed) and main bands were recovered for sequencing. Off-target efficiency was calculated with TIDE. (A-C) PAGE profiles of amplicons from candidate genome sequences. (A) PAGE profile of amplicons from HX1 wild type and knockout cells targeting *sytle5* by RNP1 and RNP 3 respectively. (B) PAGE profile of amplicons from MES1 wild type and knockout cells targeting *sytle5* by RNP1 and RNP 3 respectively. (C) PAGE profile of amplicons from HX1 wild type and gene edited cells targeting *ntrk3b* by RNP1. (D) Sequences of the candidate off-target sites and mutation efficiency of enriched bands (boxed) by PAGE.

Biology Open • Supplementary information

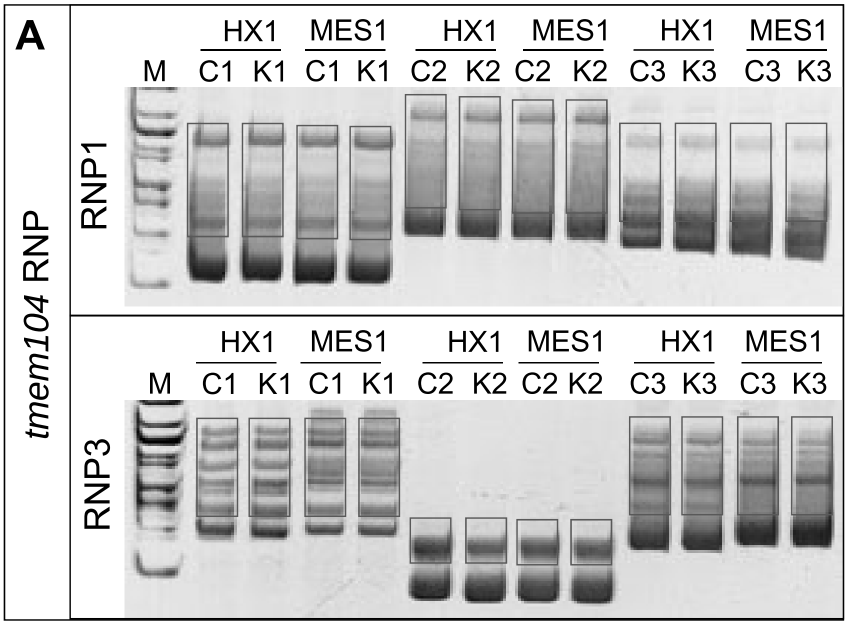

B

Sample	Off-target sequence	Enriched efficiency
tmem104-gRNA1-off1-HX1	GCCTAAACGCCTAACATGGC	1.10%
tmem104-gRNA1-off1-MES	GCCTAAACGCCTAACATGGC	3.40%
tmem104-gRNA1-off2-HX1	TACCAAACAACAAACATTGC	4.80%
tmem104-gRNA1-off2-MES	TACCAAACAACAAACATTGC	6.70%
tmem104-gRNA1-off3-HX1	TTCCCTAAGCCAAACATGCC	1.60%
tmem104-gRNA1-off3-MES	TTCCCTAAGCCAAACATGCC	2.30%
tmem104-gRNA3-off1-HX1	GTACAGTTCCNTCCTGATCG	0.00%
tmem104-gRNA3-off1-MES	GTACAGTTCCNTCCTGATCG	0.00%
tmem104-gRNA3-off2-HX1	GTACGAGATCAACCTGATGG	1.60%
tmem104-gRNA3-off2-MES	GTACGAGATCAACCTGNTGG	3.40%
tmem104-gRNA3-off3-HX1	GTGGATGATCAACCTGATCC	5.40%
tmem104-gRNA3-off3-MES	GTGGATGATCAACCTGATCC	1.40%

Fig. S2. Analysis of the enriched off-target events with PAGE and TIDE. The DNA of wild type and RNP treated cells are extracted. The off-target candidate genome sequences are selected and amplified with PCR. The DNA fragment of three off-target candidate sequence was amplified respectively from the template of HX1 and MES1 wild type control (C1-C3) and RNP knockout cells (K1-K3). After PAGE separation, enriched heteroduplex or homoduplex bands (boxed) and main bands were recovered for sequencing. Off-target efficiency was calculated with TIDE. (A) PAGE profile of amplicons from wild type and knockout cells targeting *tmem104* by RNP1. (B) Off-target sequenced and mutation efficiency of enriched bands by PAGE was shown in the table respectively.

Biology Open • Supplementary information

Table S1. Sequence of target and off-target sites

Target sequence name	MM	Target sequence [3′ sequence]	PAM	Seq ID
ntrk3b- gRNA1	0	AGCTCTAC[ACCGGACTACAG]	AGG	ENSORLG00000014606
ntrk3b-gRNA1-off 1	4	AGACCTGC[ACTGGACTACAG]	AGG	ENSORLG00000020594
ntrk3b-gRNA1-off 2	4	GGCTCTGC[TCTGGACTACAG]	TGG	XLOC_001497
ntrk3b-gRNA1-off 3	4	TCCTCTAC[ACATGACTACAG]	AGG	ENSORLG00000010913
sytl5- gRNA1	0	CCTGAACC[TCTCATTTCTGC]	TGG	ENSORLG00000008988
sytl5-gRNA1-off 1	4	CCAGACTC[CCTCATTTCTGC]	CGG	XLOC_009371
sytl5-gRNA1-off 2	4	CCCAAAAC[TGTCATTTCTGC]	TGG	XLOC_027068
sytl5-gRNA1-off 3	4	TCTTAACA[TTTCATTTCTGC]	TGG	ENSORLG00000000205
sytl5- gRNA3	0	TCAAGCAC[AAAGGCTTCCCT]	CGG	ENSORLG00000008988
sytl5-gRNA3-off 1	3	CCGAGCAC[AAAGGCTACCCT]	TGG	XLOC_003420
sytl5-gRNA3-off 2	4	TCAGGCAG[AAATGCCCTCCCT]	GGG	ENSORLG00000012755
sytl5-gRNA3-off 3	4	CCAATCAC[AGAGGCTTTCCT]	TGG	XLOC_024142
tmem104-gRNA1	0	TCCCCAAC[GCCAAACATGGC]	CGG	ENSORLG00000000869
tmem104-gRNA1-off 1	4	GCCTAAAC[GCCTAACATGGC]	TGG	ENSORLG00000006956
tmem104-gRNA1-off 2	4	TACCAAAC[ACCAAACATTGC]	AGG	ENSORLG00000003741
tmem104-gRNA1-off 3	4	TTCCCTAA[GCCAAACATGCC]	AGG	XLOC_008545
tmem104-gRNA3	0	GTACATGT[TCAACCTGATCG]	TGG	ENSORLG00000000869
tmem104-gRNA3-off 1	4	GTACAGTT[CCATCCTGATCG]	TGG	ENSORLG00000003366
tmem104-gRNA3-off 2	4	GTACGAGA[TCAACCTGATGG]	AGG	ENSORLG00000009178
tmem104-gRNA3-off 3	4	GTGGATGA[TCAACCTGATCC]	TGG	XLOC_000692

MM: number of mismatches
Target sequence: off-target sequence with highlighted mismatches in red, core in square brackets
PAM: endogenous PAM of the target site
